# Prevalence of high-risk human papillomavirus genotypes and associated factors among HIV-infected women in a reproductive age group attending antiretroviral therapy in Nekemte town, Oromia, Ethiopia

**DOI:** 10.1371/journal.pone.0357771

**Published:** 2026-09-09

**Authors:** Rundase Gebeyehu, Seifu Gizaw, Desta Amensisa, Tadesse Bekele, Belay Merkeb, Fedasan Alemu, Gadisa Cewaka

**Affiliations:** 1 Department of Medical Laboratory Sciences, College of Health Sciences, Salale University, Fitche, Ethiopia; 2 Nekemte Public Health Research and Referral Laboratory Center, Nekemte, Ethiopia; 3 Department of Pharmacy, College of Health Sciences, Salale University, Fitche, Ethiopia; Centers for Disease Control and Prevention, UNITED STATES OF AMERICA

## Abstract

**Background:**

Human papillomavirus is a deoxyribonucleic acid virus transmitted primarily sexually. Infection with high-risk human papillomavirus is recognized as the key cause of cervical cancer that is leftover as a public health problem globally. Human immunodeficiency virus infected women of a reproductive age who are on antiretroviral therapy are at higher risk due to debilitated immunity. Therefore, this study aimed to determine the prevalence of high-risk human papillomavirus and associated factors.

**Methods:**

An institutional-based cross-sectional study that included 317 HIV-infected women of a reproductive age group attending antiretroviral therapy was conducted in Nekemte town employing a systematic random sampling technique. A structured questionnaire and record reviews were used to gather the data. Cervical specimens were collected from June to August 2024 and tested at Nekemte Public Health Research and Referral Laboratory using the “Abbott real-time high-risk HPV DNA assay”. The collected data were entered into Epi-data version 3.1 and exported to the Statistical Package for Social Sciences version 26 for analysis. Descriptive statistics were used to summarize the data, and logistic regression analyses were performed to identify the determinants. The odds ratio with a 95% confidence interval was computed to assess the presence and degree of association. Following multivariable model fitting, variables with a p-value < 0.05 after adjustment for multiple testing were considered statistically significant.

**Results:**

The prevalence of high-risk human papillomavirus was 32.2% [95% CI: 27.1–37.6%], while a single genotype infection with HR-HPV-16, HR-HPV-18 and other HR-HPV accounted for 12.75%, 8.82% and 62.7% respectively. On a multivariate logistic regression, having sex before 18 years (adjusted odds ratio [AOR] =3.54, 95%CI: 1.87–6.69), multiple lifetime sex partners (AOR = 3.69, 95%CI: 1.96–6.96), and a history of sexually transmitted infection (AOR = 3.81, 95%CI: 1.93–7.51) were associated with higher odds of infection. Conversely, a longer duration on antiretroviral therapy was associated with lower odds of infection (AOR = 0.18, 95%CI: 0.06–0.54).

**Conclusion and recommendation:**

The prevalence was high in the study area. Having multiple sex partners, early sexual debut, history of sexually transmitted infections, and shorter duration on antiretroviral therapy were determinants demanding inclusive intervention efforts. Health authorities hence, need to reinforce routine human papillomavirus screening and health education for human immunodeficiency virus infected women of a reproductive age who are on antiretroviral therapy.

## Introduction

Human papillomavirus (HPV) is an infectious viral agent of papillomaviridae family that is morphologically small, circular, double-stranded deoxyribonucleic acid (DNA) of 8000 base pairs, and non-enveloped virus with icosahedral symmetry. Over 200 HPV types have been identified and more than 40 types are transmitted sexually, thus known as the genital types. Genital HPV is grouped as high-risk HPV (HR-HPV) or low-risk HPV (LR-HPV) based on capability to initiate genital cancer and tropism for epithelia [1–3].

High-risk HPV, also called oncogenic HPV has an increased affinity for the cervical mucosal lining. Infection with HR-HPV is therefore an invasion of the cervix with any of the HR-HPV genotypes that can induce malignancy resulting in cervical cancer (CC) [3,4]. The oncogenicity of HR-HPV is owing to the production of proteins called early (E) proteins (E6 and E7) which maliciously transform cervical epithelial cells [1,2].

Human papillomavirus infection is associated with cancers of different parts of our body [5]. In healthy individuals, however, about 90% of HPV infections clear within 2 years of infection while only 10% undergoes persistent infection progressing to precancerous lesions [6]. Almost 99.7% of cancers of cervix are due to persistent infection with HR-HPV [7], of which genotypes 16 and 18 are responsible for 70% making these genotypes the leading necessary causes of CC [8].

Global cancer statistics of 2020 depict that CC is the fourth-ranked cause of cancer death in women, with an incidence of 3.1 and a mortality rate of 3.4 per 100,000 women, and it is disproportionately highly incident and causes death in some countries of sub-Saharan Africa, Melanesia, South-Eastern Asia, and South America [9]. Moreover, CC cases requiring surgery are ten times higher in low and middle income countries (LMIC) despite the fact that it is a preventable disease [10]. Not only HPV, but also Human immunodeficiency virus (HIV) is highly prevalent in LMIC [11], and therefore, women in sub-Saharan African countries are burdened with both HIV and HPV infections [12].

According to the Catalan Institute of Oncology estimate of 2020, globally, more than 2.97 billion females from the general population aged ≥15 years are at risk of developing CC, with an annual new case count of 604,127 [13]. Similarly, 425.7 million women in Africa are at risk, with annual incident cases of 117,316. In the continent, there are 76,745 annual estimated death making CC the second-leading cause of cancer mortality [14]. In Ethiopia, the Global Cancer Observatory reports show the annual incidence and death from CC are 8,168 and 5,975, respectively. It is expected that the incidence will be 9,206 causing a death of 6,683 by 2025 [15].

Risk factors for HPV infection are primarily immune suppressors such as HIV, renal transplantation, long-term use of oral contraceptives, history of sexually transmitted infections (STI), and cigarette smoking [7]. The immuno-compromised groups like women living with HIV (WLHIV) are more likely to be infected due to poor HPV clearance, resulting in cervical lesions and CC. Consequently, the risk of developing CC in WLHIV is six times higher than women not infected with HIV [12,16].

Considering the urgency of the problem, the World Health Organization (WHO) calls for the implementation of global CC elimination initiatives aimed at reducing the incidence to fewer than 4 women per 100,000 women-years worldwide by 2030. The strategies are HPV vaccination, early screening, and treatment. Regular screening can detect precancerous lesions at their earliest and most treatable stages [17,18].

Despite the dual burden of HIV and HPV infection among HIV-infected women in Ethiopia, especially women in a reproductive age group, screening remained very minimal and highly dependent on cytological and histological tests which are not good indicators of HPV infections. Surveys show that screening in some parts of Ethiopia ranges from 10–27.8% [19–23], less than the WHO target (70%) [12,24]. Therefore, this study aimed at determining the prevalence and associated factors of HR-HPV infection providing insight on prevention and care of HIV-infected women of a reproductive age attending antiretroviral therapy (ART) at Nekemte town public health facilities.

## Methods and materials

### Study area and period

The study was conducted in Nekemte town from June to August 2024 at ART centers. Nekemte is the capital of the East Wollega zone, found in the western part of Ethiopia at a 327 km away from Addis Ababa, the capital town of the country. The town has seven separate self-administrative kebeles with a total population of approximately 171,994, of whom 86,899 are males and 85,095 are females. In the town, there were four public health institutions, i.e., Nekemte Comprehensive Specialized Hospital (NCSH), Wollega University Comprehensive Specialized Hospital (WUCSH), Nekemte Health Center (NHC), and Cheleleki Health Center (CHC), that provide comprehensive ART services [25].

Nekemte Comprehensive Specialized Hospital is one of the oldest government owned public health facilities found in the town which was established by Swedish missionaries in 1939. The hospital provides comprehensive preventive, curative, and rehabilitative services including ART in addition to serving as a major referral center in western Ethiopia. Similarly, WUCSH is government owned hospital established in 2017. It serves as a referral and teaching hospital under Wollega University administration. The hospital is known to provide comprehensive medical care, specialized training, and research activities across west Oromia [26,27]. Alongside those hospitals, NHC and CHC are designated governmental health centers providing key health care services for growing population of Nekemte.

Nekemte Public Health Research and Referral Laboratory Center (NPHRRL), which is one of the regional reference laboratories, is also found in the town. This laboratory serves as a specimen referral center for advanced diagnostic tests from health facilities in the western part of Oromia. It is a fully furnished laboratory performing polymerase chain reaction (PCR) tests, and the molecular laboratory department is a limited-scope accredited by fulfilling requirements of Ethiopian Accreditation services.

A total of 3,103 clients were on ART during the study period, out of which 1,706 were women of reproductive age (15–49 years), of whom 1026; 67; 518; and 95 were from NCSH, WUCSH, NHC, and CHC treatment centers, respectively.

### Study design

An institutional-based cross-sectional study was conducted at ART centers of Nekemte town public health facilities.

### Population

#### Source population.

All HIV-infected women in the reproductive age who attended ART

#### Study population.

All HIV-infected women in the reproductive age who attended ART during the study period, fulfilled inclusion criteria, signed informed consent and/or assent, and from whom cervical specimens were collected.

### Eligibility criteria

#### Inclusion criteria.

All HIV-infected women of reproductive age (15–49 years) who were on ART during the study period.

#### Exclusion criteria.

Women who were unconscious or mentally ill, on menstruation, using vaginal contraceptives or medications, undergone hysterectomy, previously confirmed to have HR-HPV, and/or treated for precancerous lesions were excluded.

### Study variables

#### Dependent variable.

- HR-HPV infection

#### Independent variables.

**Socio-demographic factors**
  - Age
  - Marital status
  - Level of education
  - Occupation
  - Residence
**Sexual behavior factors**
  - Number of sexual partners
  - Age of first sexual intercourse
**Substance use factors**
  - Smoking
  - Alcohol consumption
**Clinical factors**
  - Family planning usage
  - Number of pregnancy
  - History of abortion
  - History of STI
  - WHO clinical stage of HIV
  - HIV treatment regimen
  - Duration of ART
  - History of treatment regimen change
  - Recent CD4 count
  - Recent plasma HIV-1 viral load count

### Sample size determination

Sample size was determined using the single population proportion formula by taking (P = 35.2%) the prevalence of HR-HPV obtained from the study conducted in Shashemene town [28].


$$\begin{array}{c} n_{i}=\frac{{(Z_{\frac{\alpha }{2}})}^{2}*P(1-P)}{d^{2}}=\frac{{(1.96)}^{2}*0.352(1-0.352)}{0.05^{2}}\;=351 \end{array}$$

Since the number of study population was 1,706, the Cochran formula was used to get the corrected sample size as follows:


$$\begin{array}{c} n_{c}=\frac{n_{i}}{1+\frac{n_{i}}{N}}=\frac{351}{1+\frac{351}{1706}}=291 \end{array}$$

By adding non-response rate, the final sample size, **n**_**c**_= **321**

**Assumptions**
  - Confidence level −95%, Margin of error-5%, non-response rate-10%,

Where:

- $n_{i}$ is initial sample size from infinite population
- **N** is a sample size of finite population
- $n_{c}$ is corrected sample size
- **Z** is a critical value under standard normal distribution (1.96)
- **P** is a proportion of HR-HPV infection
- **d** is margin of error

### Sampling procedure

**Fig 1:**
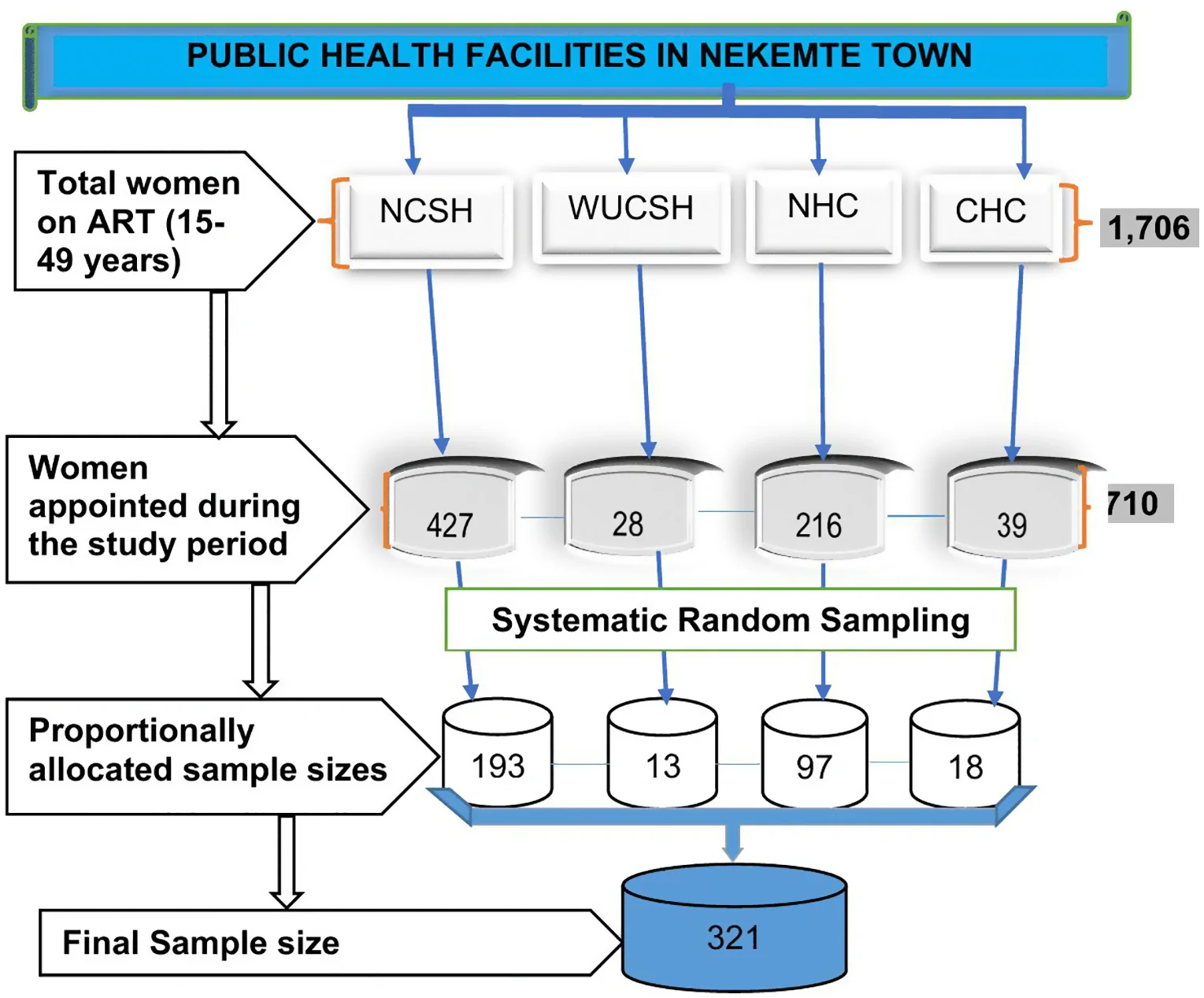
Schematic presentation of the sampling procedures at public health facilities providing ART services in Nekemte town, Oromia, Ethiopia, 2024.

A total of 1,706 HIV-infected women in a reproductive age were attending ART centers in Nekemte town public health facilities and 710 women in the age group were appointed during the study period. A systematic random sampling technique was used by computing a constant sampling interval “K” (i.e., k = 2) to get the final sample size, and a proportional number of study subjects were allocated to each ART center (Fig 1). Accordingly, a total of 321 respondents who fulfilled the eligibility criteria were selected and recruited to the study, of which 193, 13, 97 and 18 participants were from NCSH, WUCSH, NHC and CHC respectively. After explaining the purpose and procedures of the study, those who volunteer gave their consent and/or assent. Hence, every other woman of age 15–49 who attended ART centers provided cervical specimens.

### Data collection tools and procedures

#### Data collection tools.

A structured questionnaire and record review checklist were prepared to assess socio-demographic characteristics, sexual behavior, substance use, and clinical factors of participants.

#### Data collection procedures.

Data were collected by four trained and dedicated Nurses using interviewer-administered face-to-face interviews and ART clinic record reviews for clinical factors from 25/06/2024 to 24/08/2024. The records of the “length of ART” and “history of ART regimen change” were reviewed from the time participants’ therapy initiation, while the records of the “current ART regimen” and “WHO clinical stage of HIV” were obtained from registers at the time of data collection. On the other side, “the most recent CD4 count” and “recent plasma HIV-1 viral load count” records were obtained from 25/06/2023 (the most recent records in the past one-year follow-up period from the date of data collection). The information about participants was anonymous in the course of data collection or processing.

#### Cervical specimen collection, transportation and storage processes.

A single cervical swab was collected from each participant by trained and dedicated professionals at all health facilities during regular appointments of participants’ care. The request form prepared for the purpose was filled out by specimen collectors immediately. Then, the specimens were transferred to a transport tube of a collection kit. Specimens were being transported using a triple package at 2°C–30°C to the testing laboratory, NPHRRL, by one dedicated specimen runner every afternoon for storage. The testing laboratory checked sample integrity and stored specimens in a (−20°C) deep freezer. The manipulation and uncapping of the specimens were done only under BSC II to minimize HPV infections and specimen contamination [29].

#### Testing for HR-HPV and test result management processes.

This study used the Abbott real-time HR-HPV DNA assay, which employs the Abbott m2000sp instrument for processing samples and the Abbott m2000rt instrument for amplification and detection. Testing was performed by two trained medical laboratory technologists.

The primers target the most conserved L1 region of HR-HPV for amplification of the fourteen (14) genotypes of HR-HPV. The Abbott m2000sp instrument uses “_m_Sample Preparation System_DNA_” for sample preparation (extraction, concentration, and purification of target DNA molecules). The system then, captures targeted viral nucleic acid (elutes). The m2000sp also combines the elutes and amplification reagents dispensing the resulting master mix to the 96-well optical reaction plate. The reaction plate is then sealed manually by applying an optical seal [29–31]. The plate is transferred to the m2000rt for amplification and detection. Target DNA and internal control (IC) sequences are amplified simultaneously in billions by repeated thermal cycling. At the end of the amplification, the system uses fluorophores-labeled probes for detection of HPV and IC [29,30].

Probes for HPV 16, HPV 18, Other HR-HPV and IC are labeled with different fluorophores, allowing their own signals to be simultaneously detected and distinguished in a single reaction. Finally, the result is reported qualitatively as “detected” or “not detected.” If the result is detected, it can be HPV16, HPV18, other HR-HPV, or combinations of these results [30]. The results were reported to ART clinics for management. The study participants with HR-HPV positive result were managed according to “screen, triage and treat” approach and those with HR-HPV negative results were advised to return for re-screening after 2 years or when ordered by their health care provider.

### Operational definitions

**Cervical swabbing-** is a process of the collection of epithelial cells from the cervix using swabs by rotating in an anticlockwise direction three times after the opening of the cervix by speculum.

**HR-HPV infection**- A condition when the cervical specimen of the participant is confirmed to have any of the HR-HPV genotypes (HPV 16, 18, 31, 33, 35, 39, 45, 51, 52, 56, 58, 59, 66 & 68) when tested by PCR from a single specimen provided by the participant.

**Multiple sexual partner-** when a woman engages in a penetrative sexual intercourse with more than one sexual companion at once, or over a period of her life time.

**Other HR-HPV**- when a single or multiple HR-HPV of genotypes 31, 33, 35, 39, 45, 51, 52, 56, 58, 59, 66 & 68 are detected by PCR from a single specimen provided by the participant.

**Recent CD**_**4**_ **count**- The measurement/count of CD_4_ cell documented for the patient on unit ART register or ART clinic records during the most recent follow up.

**Recent HIV viral load**- The measurement of the load of HIV-1 virus in the plasma of the patient recorded on unit ART register or ART clinic records during the most recent follow up.

**Woman on ART**- A woman with known HIV infection, who is registered on ART unit register at ART clinics of the study area who were under standard antiretroviral therapy prescribed by authorized health professional.

### Quality assurance

#### Data quality assurance.

The questionnaire was prepared in the English and translated into Afaan Oromo, and it was pretested at Arjo Primary Hospital on 5% (n = 16) of the total sample size to minimize the ambiguity of words and check the applicability of the language to the local context. A final version was prepared based on the feedback obtained from the pre-test. One-day training was provided for data/specimen collectors, supervisors, specimen runner, cleaner, and testers, on the methods of extracting the pertinent data through the questionnaire, record reviews, proper specimen management and testing according to their duty in the study. Maintenance of confidentiality for the study participants was also another focus area of the training. The questionnaire was checked for consistency and completeness, and the process of data collection was overseen by two dedicated supervisors on a daily basis.

#### Laboratory quality assurance.

**Pre-analytical phase:** Reagents and supplies were used only within the expiration date, and the validity of the test kits were checked regularly. Study participants were identified by their unique ART number and a code given serially during specimen collection. Regular preventive maintenance for the instruments was performed before running the tests, and work area cleaning was carried out to prevent contamination of the specimen.

**Analytical phase:** The positive and negative controls were run and monitored with every batch of the test. The Abbott real-time HR-HPV assay detects the endogenous human beta globin sequence as an IC signal to evaluate cell adequacy, sample extraction, and amplification efficiency. A positive and negative controls were tested at every run to verify that the sample processing, the amplification, and the detection steps were performed correctly. The negative control contains only the IC sequence, which should be detected. The positive control is formulated with DNA containing HPV 16, HPV 18, HPV 58, and IC sequences, all of which should be detected [29].

**Post-analytic phase:** After testing, the results were interpreted and recorded on the report form and returned to ART centers for further management within 2 days of testing. On the other hand, specimens were retained for 2 months from the end of study period in a deep freezer (−20 °C) in case re-testing is needed. Wastes generated were managed according to institutional safety policies.

### Data processing and analysis

After collection, data was checked for completeness and consistency and entered into a pre-defined template of Epi-data version 3.1. Consequently, the data set was exported to the statistical package for Social Sciences version 26, cleaned, sorted and recoded. Out of the original sample size of 317, 12 (3.8%) participants, were excluded due to missing data, leaving 305 participants for the primary complete-case analysis. To check the robustness of the primary model, a sensitivity analysis incorporating participants with missing data was performed.

Descriptive statistics using frequency, proportions, and cross-tabulation were used to summarize the data, and the results were presented as texts, tables, and figures. Assumptions of logistic regression were assessed. Multi-collinearity was checked using the variance inflation factor and found < 5 indicating no evidence of correlation among the independent variables. Similarly, the model’s goodness of fit was tested as per Hosmer and Lemeshow’s rules and the model adequately describes out outcome variable. All explanatory variables with p-value < 0.25 on a bivariate analysis were considered as candidate variable and entered into a multivariable logistic regression model analysis. Finally, odds ratio with a 95% confidence interval was computed to assess the presence and degree of association between dependent and independent variables and the level of significance of association was determined at a P-value<0.05.

To account for multiple simultaneous statistical tests and avoid misleading conclusions from crude P-values, the Bonferroni–Holm correction was employed to minimize the family-wise error rate. When we run many statistical tests at once, the risk of getting a false positive (Type I error) increases. The Bonferroni-Holm method is an elegant statistical solution to this problem. It uses a step-down process that tests the results in order, checking each one against a customized, progressively relaxed threshold while managing the overall Type I error rate safely [32]. The steps of the method were as follows:

a) The p-values (p) were sorted from the smallest to the largest values.
b) For the i^th^ rank-ordered p-value (p_i_), the adjusted significance threshold was calculated by dividing the chosen significance level (α) by k-i + 1, where “k” represents the total number of hypotheses tested, and compared.
c) Testing was continued until we encountered a p-value that was greater than its calculated significance threshold. At that exact point, we stopped the procedure; all remaining untested hypotheses on the list were automatically declared not statistically significant.

### Ethical consideration

Ethical clearance was obtained from Salale University (SLU) Institutional Research Ethics Review Committee on 19/06/2024 according to Gregorian calendar. A written informed consents and/or assents from each study participant as well as the consents of parents/guardians of the minors were obtained. The participants were assured that the information they provided was to be used only for the study and maintained confidential. A calm conversation with participants was undertaken concerning specimen collection and laboratory result communication to minimize associated anxiety.

## Results

### Socio demographic characteristics of the participants

A total of 317 HIV-infected women were enrolled making a response rate of 98.8%. The median age of the participants was 42 years with an interquartile range (IQR) of 35–45. Majority of them, 197 (62.2%) were between 40 and 49 years and 63 (32.0%) of them were infected. Of the participants, 271 (85.5%) were urban dwellers and 173 (54.6%) were living together in a marriage whereby 31.4% of urban dwellers and 33.5% of women in marriage were infected with HR-HPV, respectively. Most participants, 130 (41%) were housewives and HR-HPV infection was comparatively higher, 8 (50%) among farmers. The majority, 106 (33.4%) attended schooling to elementary level only. The proportion of infection was the highest among who attended schooling to/above college, 11 (44%) (Table 1).

**Table 1:** Socio-demographic characteristics and HR-HPV infection among HIV-infected women in a reproductive age who were screened at ART centers of Nekemte town, Ethiopia, 2024.

Variables	Groups	Frequency (%) (n = 317)	HR-HPV Positive-n(%)
Age (years)	15-19	6(1.9)	3(50.0)
	20-24	6(1.9)	3(50.0)
	25-29	31(9.8)	11(35.5)
	30-34	33(10.4)	11(33.3)
	35-39	44(13.9)	11(25.0)
	40-44	99(31.2)	35(35.4)
	45-49	98(30.9)	28(28.6)
Residence	Urban	271(85.5)	85(31.4)
	Rural	46(14.5)	17(37.0)
Marital status	Married	173(54.6)	58(33.5)
	Divorced/widowed	119(37.5)	36(30.3)
	Single	25(7.9)	8(32.0)
Occupation	Gov’t employee	44(13.9)	15(34.1)
	House wife	130(41.0)	39(30.0)
	Merchant	79(24.9)	23(29.1)
	Farmer	16(5.0)	8(50.0)
	Daily laborer	44(13.9)	15(34.1)
	Others*	4(1.3)	2(50.0)
Education level	College and above	25(7.9)	11(44.0)
	Secondary school	75(23.7)	22(29.3)
	Elementary school	106(33.4)	36(34.0)
	Read and write only	39(12.3)	11(28.2)
	Unable to read/write	72(22.7)	22(30.6)

* Student, Female sex worker.

### Substance use and sexual behavior factors

Of the total 317 study participants enrolled, 303 (95.6%) were non-smokers and 271 (85.5%) were not consumers of any alcoholic drinks. Among smokers, 10 (71.4%) were infected by the virus. The median age of beginning penetrative sexual intercourse was 19 years with an IQR of 17–21, and 92 (29.8%) of them practiced sexual debut before their 18 years of age, where 50 (54.3%) of them were identified to harbor HR-HPV genotypes. Likewise, 135 (43.1%) respondents had multiple lifetime sex partners, 65 (48.1%) were tested positive for the virus (Table 2).

**Table 2:** Substance use, sexual behavior factors and HR-HPV infection among HIV-infected women in a reproductive age who were screened at ART centers of Nekemte town, Ethiopia, 2024.

Variables	Groups	Frequency (%) (n = 317)	HR-HPV Positive-n(%)
Smoking	No	303(95.6)	92(30.4)
	Yes	14(4.4)	10(71.4)
Alcohol consumption	No	271(85.5)	84(31.0)
	Yes	46(14.5)	18(39.1)
Age (years) of sexual debut (n = 309)	≥18years	217(70.2)	50(23.0)
	<18years	92(29.8)	50(54.3)
Number of lifetime	<2 partners	178(56.9)	34(19.1)
sex partners (n=313)	≥2 partners	135(43.1)	65(48.1)

### Clinical factors

Among the study participants, 253 (79.8%) had ever been pregnant out of which 169 (66.8%) got pregnant at least twice and a total of 70 (22.1%) women had ever experienced abortion. Among those who experienced abortion, a total of 31(44.3%) were infected. Most participants, 246 (77.6%) were using modern contraceptives, of which the majority, 101 (41.1%) and 83 (33.7%) were using injectable types and condom, respectively. Concerning the history of STIs, 74 (23.3%) reported ever contracting another STIs besides HIV; among these, 42 (56.8%) were infected by the HR-HPVs. On the other side, 293 (92.4%) attended ART for more than 2 years, where only 85(29.0%) were infected, and the median duration of ART attendance was 130 months with an IQR of 71.5–182.0. Regarding ART regimens, all participants were in the first- and second-line treatment categories and the majority, 297 (93.7%) were on the first-line drugs. Treatment regimen was ever changed for 262 (82.6%) participants in their therapeutic course (Table 3).

**Table 3:** Clinical factors and HR-HPV infection among HIV-infected women in a reproductive age who were screened at ART centers of Nekemte town, Ethiopia, 2024.

Variables	Groups	Frequency (%) (n = 317)	HR-HPV Positive-n(%)
History of pregnancy	No	64(20.2)	20(31.3)
	Yes	253(79.8)	82(32.4)
Number of pregnancy	1	84(33.2)	25(29.8)
	≥2	169(66.8)	57(33.7)
History of abortion	No	247(77.9)	71(28.7)
	Yes	70(22.1)	31(44.3)
Current contraceptive use	Non-users	71(22.4)	23(32.4)
	Condom	83(26.2)	23(27.7)
	Intra Uterine Device (IUD)	5(1.6)	2(40.0)
	Implants	21(6.6)	8(38.1)
	Injectable	101(31.9)	33(32.7)
	Pills	10(3.1)	4(40.0)
	Others^*^	26(8.2)	9(34.6)
History of STI	No	243(76.7)	60(24.7)
	Yes	74(23.3)	42(56.8)
Duration of ART	≤ 2years	24(7.6)	17(70.8)
	>2years	293(92.4)	85(29.0)
Regimen category	First line^#^	297(93.7)	94(31.6)
	Second line^$^	20(6.3)	8(40.0)
Regimen change	No	55(17.4)	21(38.2)
	Yes	262(82.6)	81(30.9)
WHO clinical stage	Stage I	303(95.6)	96(31.7)
	Others^£^	14(4.4)	6(42.9)
Recent CD_4_ count	≥500	236(74.4)	61(25.8)
(cells/µL)	<500	81(25.6)	41(50.6)
Recent HIV-1 viral	≤ 40 (TND)	291(91.8)	87(29.9)
count (copies/mL)	41-999	12(3.8)	5(41.7)
	≥1000	14(4.4)	10(71.4)

*Abstinence, calendar-based fertility control method, tubal ligation.

^#^First line fixed dose combination drugs: 1j(Tenofovir +Lamivudine+ Dolutegravir), 1e(Tenofovir+Lamivudine+Efaviranz), 1g(Abacavir+Lamivudine+Efaviranz).

^$^Second line fixed dose combination drugs: 2f(Zidovudine+Lamivudine+Ritonavir boosted Atazanavir), 2h(Tenofovir+Lamivudine+Ritonavir boosted Atazanavir), 2g (Tenofovir +Lamivudine+ Ritonavir boosted Lopinavir), 2e (Zidovudine + Lamivudine + Ritonavir boosted Lopinavir), 2i(Abacavir+Lamivudine+ Ritonavir boosted Lopinavir).

^£^WHO clinical stage II and III.

A total of 303 (95.6%) participants were in a HIV clinical stage I with HR-HPV infection of only 96 (31.7%). Participants in HIV clinical stage II and stage III accounted merely for 14 (4.4%). Similarly, the CD_4_ count recorded during the most recent follow-up indicated 236 (74.4%) respondents had a CD_4_ count of ≥500 cells/µl with a median of 706 cells/µl, and IQR of 393. From those with CD_4_ count <500 cells/µl, 41(50.6%) were infected with the HR-HPV. Likewise, HIV-1 viral load monitoring record showed that for 291 (91.8%) participants, HIV target was not detected (TND), i.e., ≤ 40 copies/ml, and 14 (4.4%) had a high HIV-1 viral count, i.e., ≥ 1000 copies/ml in their plasma. Among participants with high HIV-1 copy number, 10(71.4%) were infected with HR-HPV genotypes (Table 3).

### Prevalence of HR-HPV and its genotype distribution

**Fig 2:**
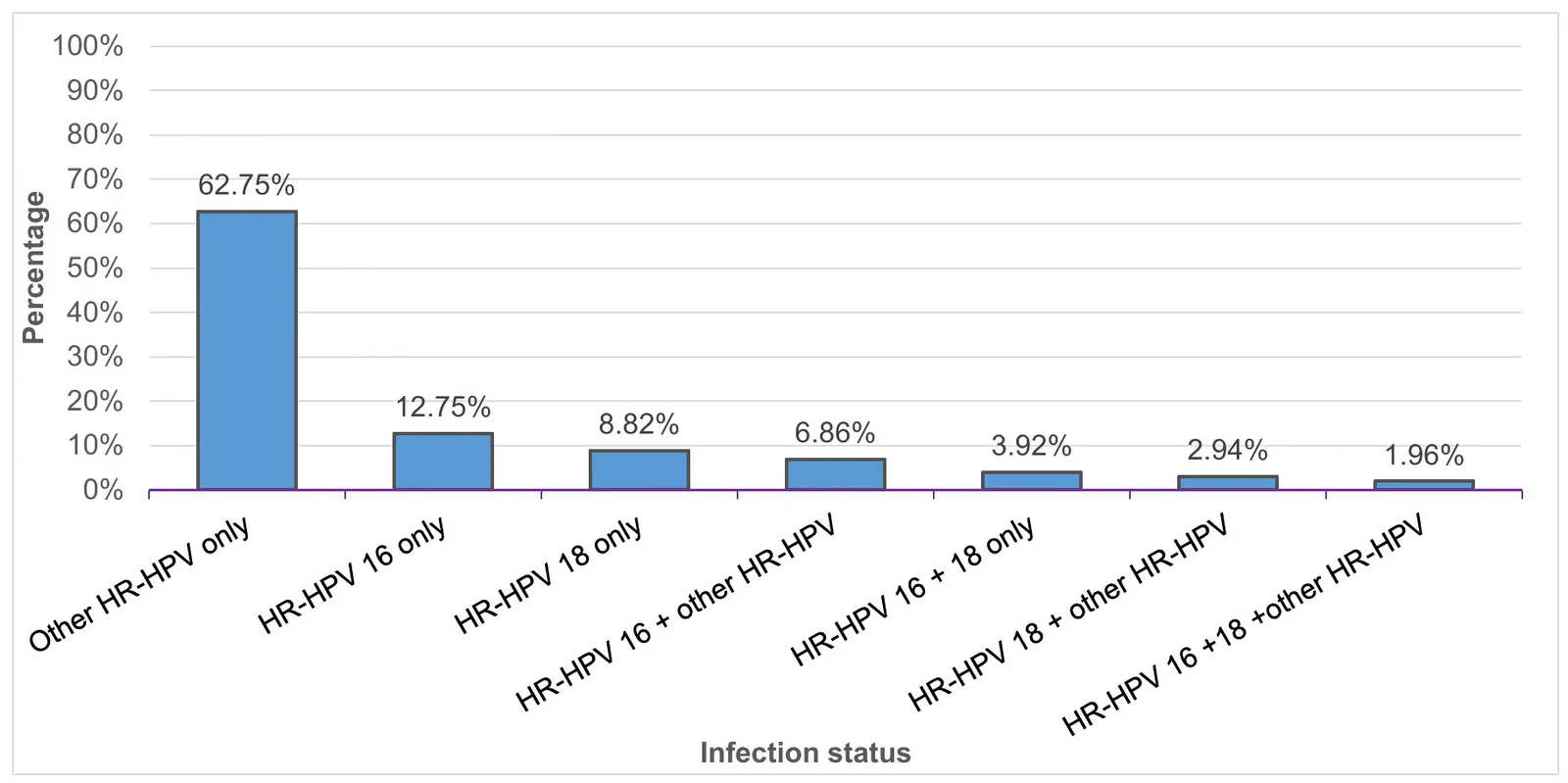
HIV-infected women in a reproductive age who were infected with various HR-HPV genotypes at ART centers of Nekemte town, Oromia, Ethiopia, 2024.

In the current study, the prevalence of HR-HPV was *32.2% [n = 102, 95% CI: 27.1–37.6]* in the study population. The most prevalent genotype, 64 (62.75%), was Other HR-HPV followed by a single infection of HPV 16 which was 13 (12.75%), and HPV 18 with a frequency of 9 (8.82%). Among 102 participants infected with HR-HPV, the genotypes known for the majority of CC (HPV 16 and/or HPV 18) were detected in 38 (37.25%) participants (Fig 2).

### Factors associated with HR-HPV infection

Bivariate logistic regression was performed for all factors, followed by multivariate logistic regression model fitting, and the results of variables associated with HR-HPV were displayed (Tables 4 and 5). Bivariate analysis was conducted to identify candidate variables at a significance level of 0.25 to be included in a multivariate logistic regression model. The level of education, smoking status, age of first sexual contact, number of lifetime sexual partners, history of abortion, history of STI, duration on ART, recent CD_4_ count, and recent plasma HIV-1 viral copy number were factors associated with the outcome variable in bivariate analysis. In contrast, no significant differences were observed between HR-HPV-infected and non-infected women by age categories, marital status, residence, occupation, alcohol drinking status, pregnancy history, pregnancy number, current contraceptive use, contraceptive types, ART regimen types, history of regimen change, and WHO clinical stages, hence, not considered as the candidate variables.

**Table 4:** Socio-demographic, substance use, and sexual behavior factors associated with HR-HPV infection among HIV-infected women in a reproductive age who were screened at ART centers of Nekemte town, Ethiopia, 2024.

Variables	Groups	AOR (95%CI)	P-value	Significance threshold
Education				
level	College and above	1		NA
	Secondary school	1.72 (0.51–5.86)	0.384	NA
	Elementary school	1.60 (0.50–5.16)	0.428	NA
	Read and write only	0.92 (0.24–3.54)	0.900	NA
	Unable to read or write	1.14 (0.34–3.88)	0.834	NA
Smokers				
	No	1		
	Yes	2.00 (0.46–8.79)	0.359	NA
Age of sexual debut				
	≥18years	1		
	<18years	3.54 (1.87–6.69)	<0.001	**0.0038**
Number of life time sex partners				
	< 2	1		
	≥ 2	3.69 (1.96–6.96)	<0.001	**0.0042**

AOR-Adjusted odds ratio, CI-Confidence interval. NA-not applicable, statistically significant P-values are in bold; Note: the model uses 305 subjects due to missing data for age of sexual debut and the number of lifetime sex partners.

**Table 5:** Clinical factors associated with HR-HPV infection among HIV-infected women in a reproductive age who were screened at ART centers of Nekemte town, Ethiopia, 2024.

Variables	Groups	AOR (95%CI)	P-value	Significance threshold
History of abortion				
	No	1		
	Yes	2.14 (1.09–4.20)	0.026	0.006
History of STI				
	No	1		
	Yes	3.81 (1.93–7.51)	<0.001	**0.0045**
Duration on ART (years)				
	≤ 2years	1		
	>2 years	0.18 (0.06–0.54)	0.002	**0.0050**
Recent CD_4_ count(cells/µL)				
	≥500	1		
	<500	2.56 (1.30–5.04)	0.006	0.0056
Recent HIV1 viral load (copies/mL)				
	≤40(TND)	1		
	41-999	0.41 (0.09–1.89)	0.251	
	≥1000	4.78 (1.09–21.03)	0.038	0.0071

**AOR**-Adjusted odds ratio, **CI**-Confidence interval. Statistically significant P-values are in bold.

Note: the model uses 305 subjects due to missing data for age of sexual debut and the number of lifetime sex partners.

In the multivariate analysis, independent variables were evaluated using the Bonferroni-Holm sequential correction to minimize the family-wise error rate across the thirteen evaluated factors, with significance defined by dynamically adjusted thresholds based on an overall α = 0.05. Variables with a P-value less than their respective sequential thresholds were considered significantly associated with HR-HPV infection. Hence, the age of first sexual intercourse (age of sexual debut), number of lifetime sexual partners, history of STI, and duration on ART were factors significantly associated with the outcome variable at the P < 0.05 level after adjustment for multiple testing. Furthermore, a sensitivity analysis incorporating participants with missing data did not meaningfully alter the adjusted odds ratios or the statistical significance of these risk factors, supporting the validity and robustness of the primary model.

Women who began sex before 18 years were more likely to be infected with HR-HPV *(AOR = 3.54, CI: 1.87–6.69, P-value < 0.001)* than those who began at/after 18 years of age. Similarly, those who had multiple lifetime sexual partners had a higher likelihood of infection *(AOR = 3.69, CI: 1.96 – 6.96, P < 0.001)* compared with women with a single-sex partner (Table 4). Moreover, those who have a history of STI other than HIV were more infected *(AOR = 3.81, CI: 1.93–7.51, P < 0.001)* than those without the history. On the other hand, participants on ART for more than 2 years had 82% lower odds of HR-HPV infection *(AOR = 0.18, CI: 0.06–0.54, P = 0.002)* than women on ART for ≤ 2 years (Table 5).

## Discussion

A pooled prevalence of HR-HPV genotypes was *32.2%* with a *95% CI (27.1–37.6).* On the other hand, early sexual engagement (before 18 years), multiple lifetime sexual partners, history of STI, and shorter duration on ART were significantly associated with HR-HPV infection.

The current study indicated that nearly a third of HIV-infected women of a reproductive age attending ART harbor genital oncogenic HPV genotypes. In fact, this notably high prevalence of HR-HPV is anticipated in developing countries like Ethiopia, where there is a limited PCR-based screening and vaccination, hence demanding a great emphasis since persistent infection with these genotypes is a proven necessary cause for CC [7]. Broadly, it implies that the CC prevention is inadequate in the study area. Even though there was a limited study conducted on WLHIV in a reproductive age group, prior research done by Monteiro et al. in a Para state of Brazil revealed a relatively higher prevalence of HPV in this age category which was 64.5% [33].

The current estimate of HR-HPV prevalence corroborates the findings of the previous studies reported among WLHIV in western Ethiopia [34], southern Ethiopia [28], Uganda [35], and Manaus of Amazonas [36], with a prevalence of 30.4%, 35.2%, 34.3%, and 31.1%, respectively. However, it is higher than that of Lome Togo [37], Zimbabwe [38], Nigeria [39], and Eastern India [40], with a corresponding prevalence of 16.7%, 24.9%, 16.3%, and 25.9%, respectively. On the contrary, the prevalence of the current study was far less than those reported in some African countries, some regions of France, Brazil, and Italy where the prevalence ranges from 40.4% to 63.3% [33,41–45].

The differences in the reported prevalence might be attributed to geographical variation, underlying health conditions of the participants, differences in sample collection and testing methods, and dissimilarities in the health care services of the countries. This high prevalence may imply that the immune dysfunction caused by HIV infection substantially increases the risk of genital HPV infection and also the persistence of the virus in the cervical cells as previously stated by other literatures [12,16,46].

Based on the results of the present study, the leading genotypes were other HR-HPV genotypes with a frequency of 64 (62.75%). Among the total study participants infected with the HR-HPV, 38 (37.25%) were known to harbor at least HPV16 and/or HPV18, the most common oncogenic genotypes linked with CC so far. Women who tested positive for HR-HPV were triaged with VIA and treatment was given to eligible women based on the “screen-triage-treat” approach. Those who were not infected with HR-HPV were advised to resume routine screening (every 2 years) according to the recommendation of the national guideline of Ethiopia unless and otherwise requested by concerned health care providers [47].

It is evident that the distribution of genotypes differs greatly from one geographical area to another, and our study showed a genotype distribution pattern that is similar with the previous studies conducted in the western part of Ethiopia [34], Ghana [43], and Nigeria [39], provided that other HR-HPV was the most prevalent, followed by HPV 16 and HPV 18 genotypes. Despite the leading prevalence of HPV 16 and HPV 18, and subsequent causation of CC specified in the previous articles [37,48–50], infection with other HR-HPV is taking dominance recently [28,34,39]. Hence, our results underscore the presence of a relatively high prevalence of other HR-HPV, which are not targeted by the widely provided bivalent vaccines.

On the other hand, there are controversies among prior literatures in establishing the relationship between independent predictors and acquisition of HR-HPV infection [28,37,42,45,51–53]. The current study indicated the odds of HR-HPV infection among women who began sexual intercourse before 18 years were nearly four times more likely to harbor the viruses than those who began at/after 18 years. It is consistent with studies done in Ethiopia [28,34], Uganda [35], and Zimbabwe [38]. However, our finding is at variance with a research conducted in South Africa [44] and Mali [42], where the age of sexual debut was not statistically significantly associated with HR-HPV infection. This variation may be due to differences in sample size, specimen collection methods and health service provision. The possible explanation of early sexual debut association with the infection could be that women who begin early sexual intercourse experience cervical trauma due to immaturity of the cervix which further escalate viral infection and the likelihood of genome integration into the host genome causing malignant transformation of cervical epithelial cells [53].

Similarly, women having multiple sexual partners (>1 partner) in their lifetime were nearly four times more likely to be infected with the HR-HPV as compared to women with a single-sex partner. Similar findings were reported from the studies done in Southern Ethiopia [28], Uganda [35] and Brazil [33]. This is an indication that the more sex partner women have, the higher the probability of contracting HR-HPV from an infected partner.

Likewise, women having a history of STI were also nearly four times more likely to harbor oncogenic HR-HPV in their cervical specimens compared with women without the history. It is in agreement with studies performed in western Ethiopia and Shashemene town in Ethiopia [28,34], Zimbabwe [54], Brazil [33], and Saudi Arabia [55]. The possible explanation is that STIs have been associated with an inflammatory response and alteration of cervical epithelial cells that could facilitate HPV infection, persistence, and consequent decline of the host’s immunity to resolve the HR-HPV infection, leading to serious complications such as cervical neoplasia [55]. Similarly, multiple genital infections also causes cervico-vaginal microbial variability which depletes *Lactobacillus species* creating a conducive environment for HPV persistence and subsequent causation of cancer [46].

With respect to ART duration, the study participants who stayed on ART for more than 2 years were 82% less likely to be infected as compared with those who were on ART for 2 years or less. Our finding agrees with a study conducted on a Malian WLHIV, which revealed that the longer the duration of ART, the less likely infection with HR-HPV [42]. Moreover, it is also similar to a study conducted in the French Antilles and Guiana that verified the protective role of ART against HR-HPV infection [41]. This suggests that long-term ART, combined with other clinical and behavioral factors, promotes immune reconstitution and mucosal defenses, thereby lowering HR-HPV acquisition and persistence rates [56].

### Limitation of the study

Since the study was based on a few health institutions we recognize that a large-scale study is needed to prepare national intervention plans and guidelines on HR-HPV infection and factors associated. On the other side, the study population may not reflect all women infected with HIV. Due to the inherent nature of the study design, it does not predict the cause-and-effect correlation between independent factors and the outcome variable. Our findings also do not indicate the incidence of infection rather than existing cases.

Similarly, the participants may not remember the past exposure accurately which may result in recall bias. Other underlying health status of participants like chronic diseases were also not studied that might have impact on variables studied. During a review of records for some clinical factors, challenges such as data inconsistencies and incompleteness were encountered. In addition, Abbott real-time HPV DNA assay can only partially genotype the viruses; hence, we were not able to completely genotype HR-HPV variants except HPV 16 and 18 genotypes.

## Conclusion

Our study highlights key epidemiological information on HR-HPV signifying the high prevalence of the virus in the study area. Other HR-HPVs, which are not targeted by bivalent vaccines, were the most prevalent genotypes detected. Our findings also identified that women with a history of early age of sexual debut, multiple lifetime sexual partners, having history of STI, and those who stayed on ART for ≤ 2 years were more likely to harbor HR-HPV than their counterparts.

### Recommendation

To meet the CC elimination target among HIV-infected women of reproductive age attending ART, health authorities and health service providers need to prioritize, integrate, and strengthen HPV DNA screening and follow-up protocols as a routine practice. Furthermore, these services should be decentralized to all ART centers alongside cytological and histological tests. Based on the findings, other HR-HPV genotypes were the most prevalent, hence, we recommend health authorities incorporate multivalent HPV vaccines, which provide broader coverage against major HR-HPV genotypes into immunization programs for women of reproductive age living with HIV who are on ART to ensure superior protection.

Moreover, routine health education is also needed for HIV-infected women of reproductive age attending ART, particularly those exhibiting the associated factors in the course of CC prevention and management. Similarly, newly diagnosed HIV-infected women should be encouraged to initiate ART immediately and maintain strict adherence; this improves immune reconstitution, thereby reducing HR-HPV infection and enhancing its clearance.

Furthermore, we recommend more studies to be conducted using rigorous study designs to identify and establish a cause-and-effect relationship between independent factors and HR-HPV infection. To have extensive knowledge of genotype distribution patterns among the study population in the region, we also recommend other researchers use complete genotyping PCR platforms in the future.

## Supporting information

S1 Data Dataset. (XLSX)

## Abbreviations

AOR: Adjusted odds ratio
ART: antiretroviral therapy
BSC II: Biosafety cabinet class II
CC: Cervical cancer
CHC: Cheleleki Health Center
CI: Confidence interval
COR: Crude odds ratio
DNA: Deoxyribonucleic acid
HIV: Human immunodeficiency virus
HPV: Human papillomavirus
HR-HPV: High-risk HPV
IC: Internal control
IQR: Interquartile range
IUD: Intra uterine device
LMIC: Low- and middle-income countries
LR-HPV: Low-risk HPV
NCSH: Nekemte Comprehensive Specialized Hospital
NHC: Nekemte Health Center
NPHRRL: Nekemte Public Health Research and Referral Laboratory Center
PCR: Polymerase chain reaction
STI: Sexually transmitted infection
TND: Target not detected
WHO: World Health Organization
WLHIV: Women living with the human immunodeficiency virus
WUCSH: Wollega University Comprehensive Specialized Hospital
